# Developments in Atomistic and Nano Structure Evolution Mechanisms of Molten Slag Using Atomistic Simulation Methods

**DOI:** 10.3390/nano14050464

**Published:** 2024-03-03

**Authors:** Chunhe Jiang, Kejiang Li, Zhisheng Bi, Shufang Ma, Jianliang Zhang, Bo Liu, Jiaqi Li

**Affiliations:** 1 Technical Support Center for Prevention and Control of Disastrous Accidents in Metal Smelting, University of Science and Technology Beijing, Beijing 100083, China; jiangchunhe@ustb.edu.cn; 2 School of Metallurgical and Ecological Engineering, University of Science and Technology Beijing, Beijing 100083, China; bizhisheng_ustb@126.com (Z.B.); zhang.jianliang@hotmail.com (J.Z.); 3 Aetna Tianlong Tungsten Molybdenum Technology Co., Ltd., No. 11 Fenghui Middle Road, Haidian District, Beijing 100094, China; mashufang03@163.com; 4 School of Chemical Engineering, The University of Queensland, Saint Lucia, QLD 4072, Australia; 5 School of Advanced Engineering, University of Science and Technology Beijing, Beijing 100083, China; 13979217343@163.com (B.L.); U202240410@xs.ustb.edu.cn (J.L.)

**Keywords:** molten slag, molecular dynamic, nano structure, atomistic structure

## Abstract

Molten slag has different properties depending on its composition. The relationship between its composition, structure, and properties has been the focus of attention in industrial manufacturing processes. This review describes the atomistic scale mechanisms by which oxides of different compositions affect the properties and structure of slag, and depicts the current state of research in the atomic simulation of molten slag. At present, the research on the macroscopic properties of molten slag mainly focuses on viscosity, free-running temperature, melting point, and desulphurization capacity. Regulating the composition has become the most direct and effective way to control slag properties. Analysis of the microevolution mechanism is the fundamental way to grasp the macroscopic properties. The microstructural evolution mechanism, especially at the atomic and nanoscale of molten slag, is reviewed from three aspects: basic oxides, acidic oxides, and amphoteric oxides. The evolution of macroscopic properties is analyzed in depth through the evolution of the atomic structure. Resolution of the macroscopic properties of molten slag by the atomic structure plays a crucial role in the development of fundamental theories of physicochemistry.

## 1. Introduction

Molten slag is a byproduct formed in the metallurgical process when oxide melts are involved. In the metallurgical process, slag serves a crucial role by facilitating the separation of gangue from liquid iron. Additionally, harmful impurities like phosphorus and sulfur can be effectively eliminated through the careful adjustment of slag compositions. Moreover, slag acts as a protective barrier, shielding the liquid steel from direct contact with elements such as hydrogen, nitrogen, and oxygen. Its multifaceted functions are pivotal in ensuring product quality, optimizing iron recovery rates, maintaining stable smelting operations, and achieving various technical and economic benchmarks [1,2,3,4,5].

Common oxides in molten slag can be divided into three types, including acid oxide, basic oxide, and amphoteric oxide [6,7]. As a representative of acidic oxides, SiO_2_ is a strong network former, and its atomic structure basically exists as a [SiO_4_]^4−^ tetrahedron. SiO_2_ can increase the stability and polymerization degree of slag due to its network formation ability. It requires higher energy to destroy the network structure with [SiO_4_]^4−^ as the basic unit. In addition, P_2_O_5_ is also a common acid oxide in aluminosilicate melt. CaO is a typical basic oxide and the main component for adjusting basicity. Its content has a vital impact on desulfurization and dephosphorization. In addition, increasing the content of CaO can improve the viscosity of slag. The properties of MgO are similar to CaO, and it can also improve fluidity by reducing the viscosity of the slag [8,9]. Al_2_O_3_ is a representative amphoteric oxide, and there are obvious differences in its properties in different contents. Excessive Al_2_O_3_ generally leads to an increase in slag viscosity, playing the role of acid oxide [10]. When the basic oxide is insufficient, Al_2_O_3_ will play the role of a basic oxide to ensure the balanced charge of the system [11]. B and Al elements belong to the same main group; they often have similar physical and chemical properties [12,13]. B_2_O_5_ can reduce the viscosity and the melting point of slag. In the micro scale, B^3+^ ions can increase the polymerization degree of the slag system. However, B_2_O_3_ has the ability to reduce slag viscosity [14,15]. Therefore, the adjustment mechanism of B_2_O_3_ on the properties of slag is worthy of further fundamental research. TiO_2_ usually has amphoteric properties [16]. In addition to the above main ingredients, due to the impact of ore resources, it may also contain a small number of other compounds, such as K_2_O, Na_2_O, MnO, FeO, etc.

The research on the macroscopic properties of slag is mainly focused on aspects such as melting characteristics, temperature, and fluidity [17,18,19,20]. Over the decades, a considerable amount of research has provided a comprehensive understanding of the high-temperature properties of slag. However, due to the limitations of high-temperature experiments, observing the microscopic structure has become a challenging aspect in the field of experimental research. With the development of modern physical chemistry and computational science, molecular dynamics simulation has become an effective method for researchers to study nanostructures under extreme conditions. Since the 21st century, molecular dynamics simulation methods have gradually been introduced into the field of metallurgy. Research on molten slag is the most widely applied application of molecular dynamics in metallurgy [21]. Through molecular dynamics simulation, researchers have vividly defined the types of oxygen atoms, characterized the network structure, and depicted the evolution of atomic properties. The transition from low-temperature qualitative characterization to high-temperature quantitative research has been achieved. Analyzing the microstructural evolution mechanisms of slag is crucial for controlling its macroscopic properties. Currently, traditional molecular dynamics can simulate studies on oxides such as SiO_2_, Al_2_O_3_, CaO, MgO, TiO_2_, P_2_O_5_, Na_2_O, K_2_O, B_2_O_3_, FeO, Fe_2_O_3_, MnO, etc. Basic microscopic properties, including radial distribution functions; coordination numbers; microscopic structural units; diffusion coefficients; etc., can be accurately computed. However, there is still a significant lack of information on more in-depth electronic structures. Meanwhile, classical molecular dynamics does not have the ability to simulate trace components in slag due to the limitations of potential parameters. Although first-principles molecular dynamics can study all elements, it currently cannot achieve the efficiency of molecular dynamics simulation due to its strong dependence on computational resources. Machine learning molecular dynamics is addressing both accuracy and computational efficiency issues, and is currently being applied to slag research.

This paper aims to review the research progress of molten slag, especially the relationship between the nanostructure and the macroproperties. Clarifying the action mechanism of oxides on the slag is critical to guiding the metallurgical process and the production process of other silicate-related industries.

## 2. The Atomic Simulation Method

**Figure 1:**
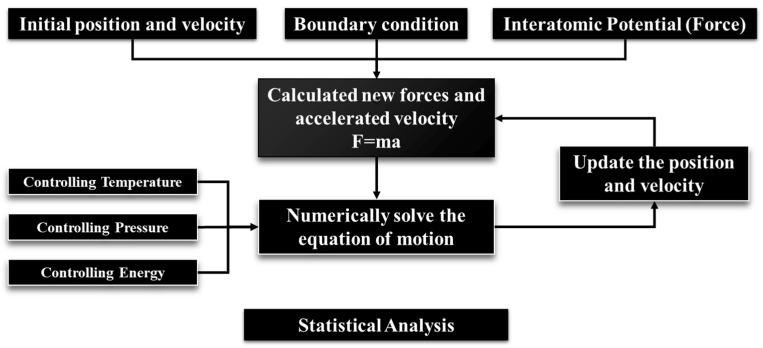
Schematic diagram of molecular dynamics simulation.

Molecular dynamics simulation is a branch of computational chemistry based on Newtonian classical mechanics. It calculates the trajectories of atoms by computing the interatomic forces, and employs statistical methods to analyze the thermodynamic/dynamic properties of the system. The fundamental steps of molecular dynamics are illustrated in Figure 1. Through the analysis of atomic trajectories, information such as the structure, energy, thermodynamics, and mechanics of the system over a period of time can be obtained. This allows researchers to obtain desired computational results tailored to their specific research directions.

Molecular dynamics methods, based on different approaches to handling interatomic forces, can be categorized into classical molecular dynamics (CMD); ab initio molecular dynamics (AIMD); and machine learning molecular dynamics (MLMD). CMD typically employs a classical potential energy function expressed in a fixed mathematical form to describe the interatomic forces, while AIMD calculates interatomic interactions through quantum chemistry methods. MLMD, on the other hand, utilizes machine learning techniques to train ab initio simulation results to obtain a machine-learned potential. Although these three methods aim to describe interactions differently, they ultimately analyze the physical and chemical properties of a system based on the motion patterns of atoms.

### 2.1. Classic Molecular Dynamic

In CMD simulations, the choice of the force field is a crucial factor determining the accuracy of the simulation. The form of the force field is often fixed, generally describing the interactions of atoms based on the atomic distance and location. For these different categories of descriptions, classical force fields widely applied in CMD simulations currently include Lennard–Jones (LJ); Born–Mayer–Huggins (BMH); the embedded atom method (EAM) [22]; CHARMM [23]; AMBER [24]; ReaxFF [25]; and others. The emphases of these force fields varies based on the characteristics of different systems.

CMD is the primary method used for studying the atomistic structure of molten slag, as outlined in Table 1. BMH is the main force field employed in slag research, accurately describing the interactions between atoms, and thereby resolving atomic structural relationships. Tersoff [26] appears specifically relevant in the description of SiO_2_ single crystals. COMPASS [27] is predominantly utilized in molecular simulations using Materials Studio software. MORSE is mainly used in conjunction with the BMH to correct short-range interactions. Current CMD researches on slag have broadly covered common components. However, research on trace elements such as BaO, Cr_2_O_3_, etc., is still lacking, primarily due to the absence of force fields or parameters that can accurately describe their interatomic interactions.

### 2.2. Ab Initio Molecular Dynamics

Compared to CMD, AIMD can compute potential energy and forces using quantum chemistry methods [92,93]. It solves the Schrödinger equation with different kinds of approximations and then determines the trajectories of atomic motion based on Newton’s second law. Since each step in AIMD involves quantum chemical calculations, it heavily relies on computational resources, and the obtained simulation results are typically in the picosecond range. However, AIMD’s advantage lies in its independence from the choice of force field. It can accurately describe interatomic interactions even for less common elements, enabling precise simulations.

With the continuous development of computer technology, AIMD has gradually been applied to slag research, as shown in Table 2. AIMD can not only produce results similar to CMD, but also provide additional information at the electronic scale, such as charge distribution and band structure. Currently, there are relatively few research achievements using AIMD, but it is expected to become an important method in future researches.

### 2.3. Machine Learning Molecular Dynamics

Machine learning is not a new concept in the field of natural sciences [104], and its primary applications include material structure prediction [105], new material development, and material discovery [106]. However, in the field of molecular dynamics, machine learning is primarily used to obtain accurate interatomic interaction potentials. Machine learning potentials can, on one hand, compensate for the dependence of CMD on parameterized empirical force fields, and on the other hand, address the unacceptable computing resource consumption of large-scale AIMD simulations. MLMD involves training on energy and force information obtained from DFT calculations [107]. The generation of a machine learning potential typically requires three essential steps: (1) a dataset consisting of reference structures and corresponding quantum mechanics (QM) information; (2) a descriptor of the atomic neighbor structure information, which can be input into the machine learning algorithm; (3) a regressor to fit a multilayer perceptron. Based on quantum chemical descriptions and the extension of machine learning, MLMD has become another hot research direction in molecular dynamics simulations.

AIMD, due to its excessive dependence on computational resources, results in smaller system simulations and shorter simulation times compared to CMD. MLMD, on the other hand, allows for easy enlargement of the system, better capturing of accidental events during simulations, and thus the discovery of special phenomena in nature. In slag systems, MLMD has already found practical applications [108]. By thoroughly analyzing large-scale molecular dynamics data, machine learning algorithms can uncover microscopic features hidden in slag, providing a more comprehensive understanding of slag properties. The performance of MLMD is limited by the quality and quantity of training data. Obtaining a high-quality model requires a significant amount of real and diverse molecular dynamics data, which can be a substantial challenge.

## 3. The Properties of Molten Slag

**Figure 2:**
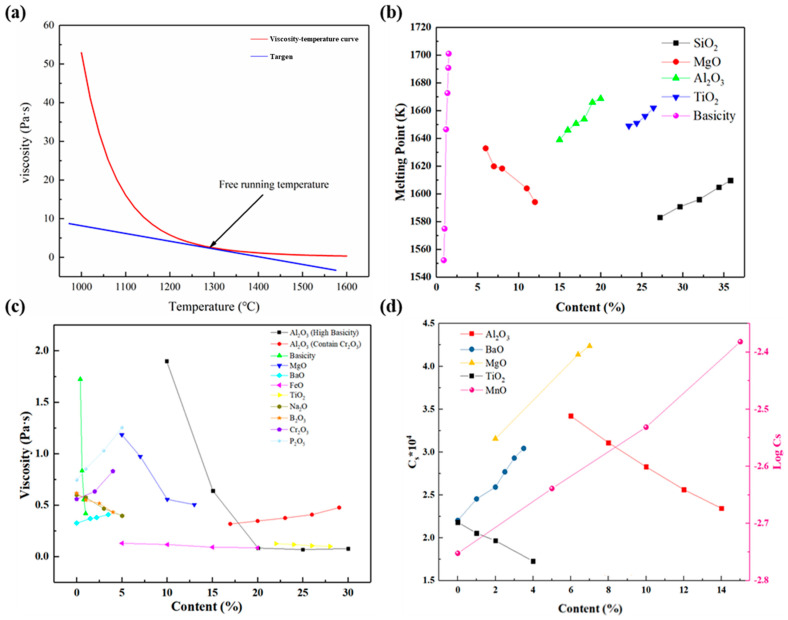
(**a**) The viscosity–temperature curve of slag ([110,111]); (**b**) the evolution of melting point with different compositions ([10,112,113]); (**c**) the variation of viscosity with different content ([114,115,116,117,118,119,120,121,122,123,131]); (**d**) the evolution of sulfur capacity with different contents ([126,127,128,130,131]).

The properties of slag are closely related to its chemical composition, which generally directly affects its performance—such as the melting point, free running temperature, viscosity thermal conductivity, desulfuration ability, etc. The melting point and free running temperature are two important indicators used to evaluate the slag melting process [109,110]. The free running temperature means that although the slag has not reached the melting point, the viscosity has reached a certain threshold, and the slag can flow freely. It is defined according to the viscosity (Pa·s)-temperature (°C) curves, as shown in Figure 2a. The procedure for calculating entails drawing a tangent line with a slope of -0.02, with the temperature at the tangent point representing the slag’s free-running temperature [109,110], while the melting point refers to the temperature that the object melts completely from solid state to liquid. As shown in Figure 2b, the composition of slag directly influences the variation in slag’s melting point, which is directly related to the alteration of slag’s microstructure. The acid oxides represented by SiO_2_ can increase the melting point of the slag due to their strong network formation capabilities [111]. However, the effect of basic oxides is exactly the opposite, and within a certain range of compositions, they can often reduce the melting point of slag [111]. In addition, the amphoteric oxides, such as Al_2_O_3_, also have the ability to increase the melting point of slag [10,112]. Therefore, to fully understand the changes in macroscopic properties, it is necessary to conduct an in-depth analysis from the microstructure of the slag.

In general, the operating temperatures of metallurgical processes exceed the melting point of slag, making the fluidity of slag a key parameter of concern. During metallurgical processes, viscosity changes are typically controlled by adjusting the composition of slag. However, as high-quality mineral resources become increasingly scarce, the variety of ores entering the furnace has become richer, resulting in complex compositions of slag. In recent years, there has been extensive research on the impact of changes in different oxide components on slag viscosity, as shown in Figure 2c. Generally, basic oxides can improve slag fluidity by reducing the structural complexity. Previous research has already demonstrated that basic oxides, such as CaO [113], MgO [114], FeO [115], Na_2_O [116], etc., possess this characteristic. However, if the content is too high, there is a possibility of increasing the melting point, consequently leading to a decrease in the fluidity of slag. Xing et al. has found that the addition of BaO to slag leads to the formation of more complex structure, thereby increasing the viscosity. Acidic oxides generally only play the role of increasing structural complexity in slag. Therefore, SiO_2_ and P_2_O_5_ [117,118,119,120] will directly cause the viscosity of the slag to increase, while the role of amphoteric oxides will have different effects on the fluidity of slag according to changes in relative composition and temperature. Figure 2c shows that the influence of Al_2_O_3_ on the viscosity of slag is obviously different under different composition conditions [121]. The roles played by amphoteric oxides such as Cr_2_O_3_ [122] are similar to those of Al_2_O_3_. Therefore, to clarify the mechanism of action of amphoteric oxides, detailed analysis is required based on different conditions.

During the metallurgical process, changes in slag properties are crucial to the adjustment of impurity elements in molten metal. The desulfurization process is the most intuitive process of regulating slag properties. Sulfur is a harmful impurity in steel products. Its presence in steel materials will seriously affect their macroscopic properties. Therefore, regulating the sulfur content in materials is crucial to the production of high-quality steel materials [123]. In thermodynamics, sulfur capacity is usually used to characterize the desulfurization ability of slag, as shown in Figure 2d. Public researches showed that basic oxide components, such as CaO [124], MgO [125], MnO [126], and BaO [127] can improve the desulfurization ability of slag, while components such as SiO_2_ [128], Al_2_O_3_ [129], and TiO_2_ [130] have the opposite effect. The sulfur capacity of slag is also directly related to its microstructure evolution [125,126,127,129,130].

During the metallurgical process, regulating the composition of slag is the most intuitive method to adjust the evolution of the macroscopic properties of slag. In current research, most of the composition changes have been investigated in detail. However, high-temperature experiments are dangerous and complicated, and current research methods cannot detect the microstructural variation of slag under high-temperature conditions in real time. Therefore, the microscopic analysis of slag is still lacking. Developments in science have proven that microstructure is the fundamental factor affecting the evolution of macroscopic properties. Moreover, the ingredient adjustment method summarized through experience lacks scientificity. Therefore, analyzing the microstructure evolution mechanism of slag with different components is crucial to understanding the evolution of properties.

## 4. The Evolution of Molten Slag’s Microstructure

**Figure 3:**
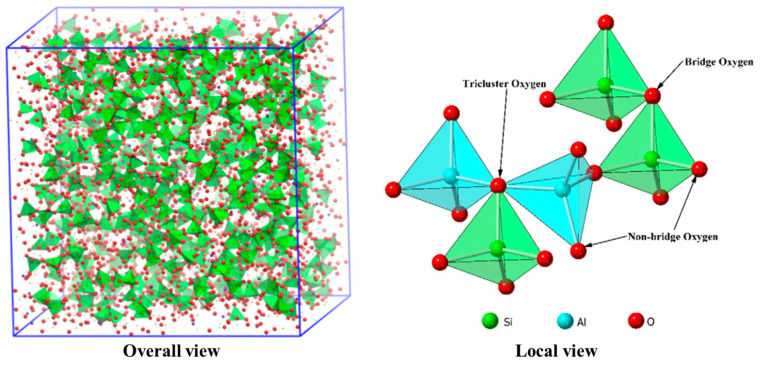
Atomic configuration of oxygen structural unit (Reproduced with permission from [64]. Elsevier, 2020).

Molten slag is mainly composed of aluminosilicate, consisting of [SiO_4_]^4−^ and [AlO_4_]^5−^ tetrahedron. In the nanoscale network structure, the existence of oxygen atoms is critical to the properties of the aluminosilicate system. According to the role of oxygen, as shown in Figure 3, oxygen atoms can be divided into four types: free oxygen (FO), non-bridge oxygen (NBO), bridge oxygen (BO), and tricluster oxygen (TO). The network structure of molten slag is mainly connected by BO [53,64]. BO refers to the oxygen atom that connects the two tetrahedron structures, typically Si-O-Si, Si-O-Al, or Al-O-Al. Generally, three tetrahedron structures are connected by TOs, which is usually less in the system, but also plays an important role in improving the degree of polymerization (DOP) of the system. The oxygen atom occupied by only one tetrahedron structure is NBO, which exists only at the edge of the tetrahedron structure. The higher the NBO content, the lower the DOP of the system. In addition, there will be some FOs in the system. As usual, the ratio of BO to NBO can be used to represent the DOP of the system. Moreover, the number of BO on the tetrahedron can also represent the DOP of the system, such as Q_Si_^n^ and Q_Al_^n^ [42] (the proportion represented by SiO_4_ or AlO_4_ tetrahedra with non-bridging oxygen atoms). DOP represents the complexity of the microstructure of the slag. The more complicated the structure, the more difficult it is to change its original properties, and the more stable. DOP is directly related to the properties, such as viscosity, melting temperature, thermal conductivity, and desulfurization capacity, of molten slag.

### 4.1. The Effect of Acid Oxides

**Figure 4:**
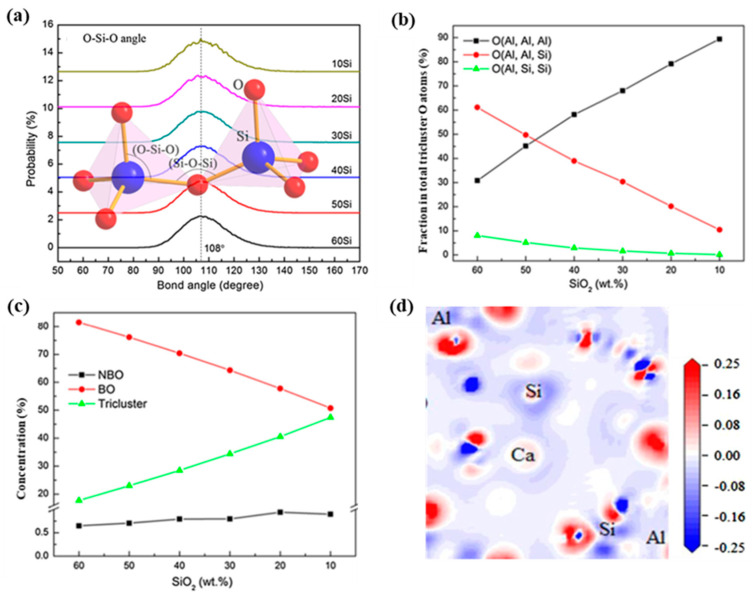
(**a**) Distribution of bond angle of O-Si-O with change of SiO_2_ content (Reproduced with permission from [30]. Elsevier, 2016); (**b**) evolution of non-bridging oxygen, bridge oxygen and tricluster oxygen with change of SiO_2_ content (Reproduced with permission from [30]. Elsevier, 2016); (**c**) fraction of different types of O triclusters amongst total triclusters with change of SiO_2_ content (Reproduced with permission from [30]. Elsevier, 2016); (**d**) electron density difference calculated at 1773 K (Reproduced with permission from [96]. Elsevier, 2020).

SiO_2_ and P_2_O_5_ are common acidic oxides that make up the tetrahedron structure in the slag. Usually, SiO_2_ is the main component in metallurgical molten slag, and P_2_O_5_ only exists in small amounts. In the silicate system, the bond length of the Si-O is about 1.67Å [101]. Si mainly forms a tetrahedron structure with 4 O atoms. The bond angle of O-Si-O is generally maintained at 108°, as shown in Figure 4a [28]. Si is the atom with the smallest diffusion coefficient in the slag, and the worst movement. Li et al. [30] investigated the influence of SiO_2_ content on the local structure and viscosity of slag. It was found that the main structural unit of the formation of the Si-O network was a [SiO_4_]^4−^ tetrahedron. As shown in Figure 4b,c, with the decrease of SiO_2_ the BO content decreases, while the TO increases, in which the amount of O(Al, Al, Al) increases and the amount of O(Al, Al, Si) and O(Al, Al, Si) decreases. It indicates that [AlO_4_]^5−^ tetrahedra are more likely to be connected by TO, while [SiO_4_]^4−^ tetrahedra prefer to be connected by BO. Due to the stable structure of a [SiO_4_]^4−^ tetrahedron, the chemical composition has little effect on the bond length of Si-O. With the decrease of SiO_2_ content, the diffusion coefficients of Si, Al, Ca, and O atoms increase significantly, which is mainly because the reduction of the Si-O bond weakens the stability of the network structure. Therefore, the viscosity of the system is reduced, which proves that the acid oxide has an obvious effect on the fluidity of the system. Sajid et al. investigated the electronic structure of SiO_2_-based slag, as shown in Figure 4d. Oxygen atoms typically gain more electrons, while Si and Al atoms primarily lose electrons, resulting in the formation of Si-O and Al-O bonds. Due to the greater electron loss by silicon atoms, the Si-O bond is the most stable chemical bond in the furnace slag system [96].

**Figure 5:**
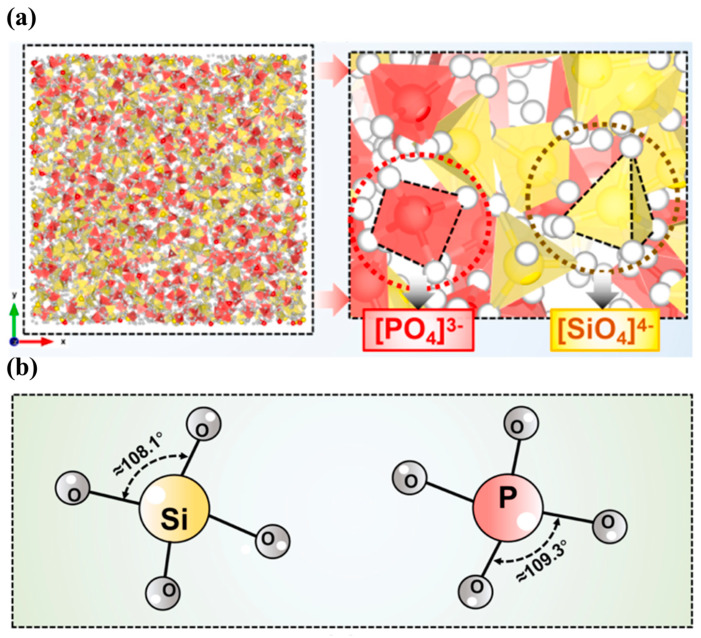
(**a**) Silicate network and phosphate network structure in molten slag system (Reproduced with permission from [77]. Elsevier, 2024); (**b**) schematic diagram of average bond angles of O–Si–O, and O–P–O (Reproduced with permission from [72]. J-STAGE, 2015).

P_2_O_5_ is an acidic oxide with a lesser content in the molten slag [76], and it can also act as the network former in the slag, as shown in Figure 5a [77]. The bond length of P-O is about 1.53Å [48], which is slightly shorter than the Si-O. The stability of the P-O bond is relatively weaker than the Si-O bond. Fan et al. [72] found that the bond length of P-O will decrease as the P_2_O_5_ content increases. P atoms are more inclined to form an almost perfect [PO_4_]^3−^ tetrahedron structure. The bond angle of O-P-O is about 109.3°, slightly larger than that of O-Si-O, as shown in Figure 5b. Du et al. [75] explored the microstructure of different P_2_O_5_-based binary melt. The basic unit of the P_2_O_5_-based binary melt is a [PO_4_]^3−^ tetrahedron, which is very close to the perfect tetrahedron structure. With the increase in P_2_O_5_ content, Q^0^, Q^1^, and Q^2^ will gradually convert to Q^3^ structure, resulting in the more complex network structure of each phosphate system. Qian et al. [98] found that the role of P_2_O_5_ in aluminosilicate varies with the content. When the P_2_O_5_ content is minimal, most of the O atoms in [PO_4_]^3−^ are NBOs, which can play a role in repolymerizing a [SiO_4_]^4−^ based network structure and promote the increasing of the Si-O-Si proportion. With the further increase in the P_2_O_5_ content, the number of BO in the [PO_4_]^3−^ tetrahedron structure continue to increase, and P atoms gradually participate in the construction of the network structure.

B_2_O_3_ is an excellent surface-active substance which can reduce the viscosity of slag. As a flux, B_2_O_3_ can be substituted for CaF_2_ to reduce the free-running temperature [15]. Structurally, B_2_O_3_-containing slag is more likely to form [BO_3_]^3−^ trigonal planar structures, which affects the structure and properties of the slag. Wang et al. [132]. demonstrated that B_2_O_3_ has a significant inhibitory effect on CaO-SiO_2_ activity. Within a certain content, increasing the B_2_O_3_ content can reduce the strength of the network structure, and decrease the slag viscosity and apparent activation energy. Lai et al. [133] found that B_2_O_3_ leads to the formation of [SiO_4_]^4−^ structures that increase the polymerization of a CaO-Al_2_O_3_-SiO_2_-TiO_2_-B_2_O_3_ system. The B and O atoms mainly form two structures, [BO_3_]^3−^ trigonal planar and [BO_4_]^5−^ tetrahedron. Zhang et al.’s research [83] found that the addition of B_2_O_3_ results in the formation of a B-O network structure, which becomes randomly mixed with the initial Si-O network structure. However, the properties exhibited by B_2_O_3_ vary significantly under different slag conditions. In low basicity conditions, B_2_O_3_ reduces the slag’s polymerization, whereas in high basicity conditions, the opposite occurs. Bi et al. [35] further revealed that B_2_O_3_ can exhibit amphoteric properties, like Al_2_O_3_. In the SiO_2_-CaO-B_2_O_3_ system, it transforms from acidic to basic when the B_2_O_3_ content is greater than 21%. When the content of acidic oxides is too high, B_2_O_3_ will show basic properties, but the role played is completely different from Ca^2+^. The Ca ions reduce the system’s polymerization by destroying the BO, while B^3+^ tends to capture O^2+^ to form chemical bonds. In turn, it changes from decreasing the molten slag microstructure polymerization, to reducing the slag complexity [37]. However, generally molten slag has a low content of B_2_O_3_, which can only show the nature of acidic oxides.

### 4.2. The Effect of Basic Oxides

**Figure 6:**
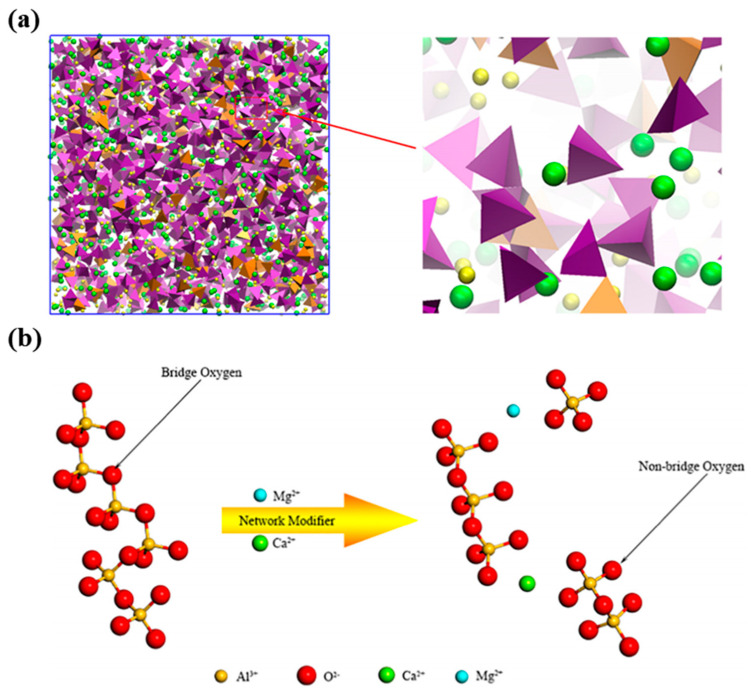
(**a**) Structure of molten slag (purple tetrahedron represents [SiO_4_], orange tetrahedron represents [AlO_4_], green and yellow balls represent Ca and Mg cations, respectively) (Reproduced with permission from [40]. Elsevier, 2019); (**b**) depolymerization of network structure with MgO and CaO (Reproduced with permission from [52]. Elsevier, 2018).

CaO and MgO are the most common basic oxides in metallurgical molten slags, and are generally used as network modifiers to adjust the macroscopic properties. Microscopically, CaO and MgO dissociate into Ca^2+^, Mg^2+^, and O^2−^, as shown in Figure 6a. The cations will interact with the network structure formed by the acidic and amphoteric oxides in the slag, thus affecting the macroscopic properties of the slag. Jiang et al. [40,41] comparatively analyzed the mechanism of microscopic effects of CaO and MgO on the slag properties. The results show that the changes in the relative contents of CaO and MgO do not affect the interatomic chemical bond length in the slag. However, Ca^2+^ and Mg^2+^ ions are playing the role of network modifiers, which will damage the intrinsic network structure, as shown in Figure 6b. The increase in CaO content will lead to more conversion of the TO into BO and NBO, which in turn reduces the structural complexity of the system. MgO has the same function of reducing the structural complexity of the slag. Nevertheless, when CaO and MgO are only present alone and in the same amount, MgO is significantly more capable of destroying the microscopic network structure. Ca^2+^ ions have a stronger charge compensation ability than Mg^2+^ ions, so Ca^2+^ is more effective in maintaining the stability of the network structure. Meanwhile, both ions do not significantly affect the bond angles of the network structure. Zhang et al. [100] utilized AIMD simulations to discover that only a small amount of electron accumulation occurs around Ca atoms. Therefore, Ca-O is the ionic bond in the molten slag. Gao et al. [114] further demonstrated by Fourier transform infrared (FTIR) spectroscopy that the complex network structure of slag can be depolymerized into simpler units by CaO or MgO.

**Figure 7:**
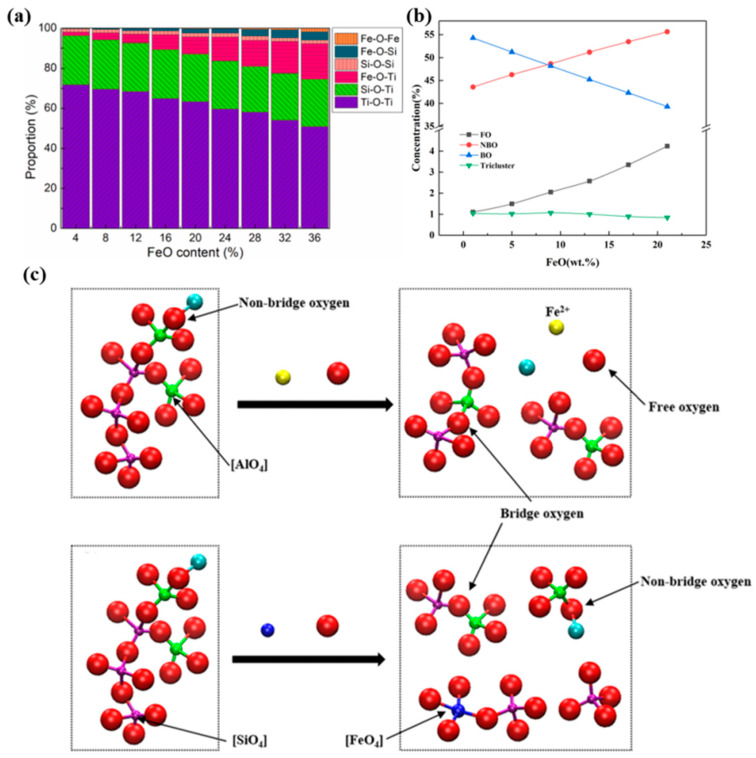
(**a**) The oxygen connection state in FeO-based slag with varying FeO content (Reproduced with permission from [57]. Elsevier, 2021); (**b**) the evolution of bridging oxygen, non-bridging oxygen, tricluster oxygen and free oxygen with the FeO content (Reproduced with permission from [58]. Springer Nature, 2021); (**c**) a schematic diagram of local atomic structure, which is also used to explain the variation of BO, NBO, FO, and TO in the system (Reproduced with permission from [59]. Springer, 2022).

FeO is a special component of molten slag, which both dissolves into the slag to influence the evolution of slag properties, and changes the slag-iron interaction through reduction process. Macroscopically, FeO has the ability to reduce slag viscosity. Fan et al. [57] investigated the effect of FeO on the structure and transport properties of the TiO_2_-SiO_2_ slag system and found that FeO has the ability to destroy the Ti-O-Ti structure. The Fe has the effect of reducing the complexity of the slag by replacing one of the Ti ions in the structure, which in turn improves the fluidity, as shown in Figure 7a. Ma et al. [58] further investigated the effect of FeO on the SiO-Al_2_O_3_-CaO slag system, and further resolved that FeO can improve the slag by promoting the conversion of TO and BO to NBO, as shown in Figure 7b. However, CaO destroys the network structure faster than FeO under the same condition [45]. Fe atoms can exist in the slag in both Fe^2+^ (FeO) and Fe^3+^ (Fe_2_O_3_) ions. It was found that both Fe^2+^ and Fe^3+^ have the ability to reduce the BO, and the effect of Fe^3+^ is relatively stronger. Fe^3+^ can form the FeO_4_ structure by replacing Si or Al atoms, which in turn has the effect of reducing the degree of polymerization, as shown in Figure 7c. Fe^2+^, on the other hand, tends to exert only a similar effect as Ca^2+^ and Mg^2+^ [59].

**Figure 8:**
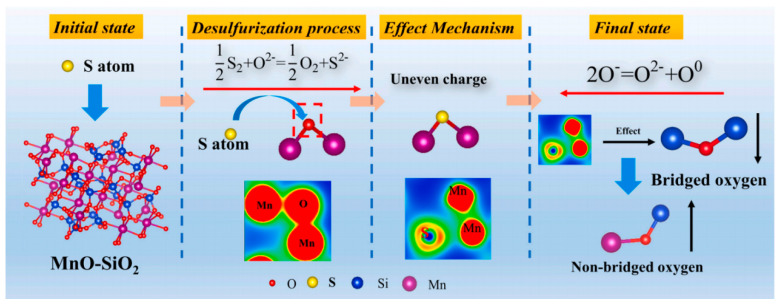
Schematic diagram of desulfurization mechanism of liquid MnO-SiO_2_ system (Reproduced with permission from [99]. Elsevier, 2022).

With the shortage of advantageous mineral resources, the utilization of multicomponent minerals gradually leads to composition diversification in metallurgical molten slag. MnO is increasingly becoming a more common component. As expressed previously, MnO has the ability to improve slag viscosity and enhance desulphurization. Ma et al. [71] further investigated the mechanism of the effect of MnO on the slag under high temperature conditions. It was found that the change in MnO content does not directly affect the bond length of the original chemical bond, but it leads to a decrease in the coordination number of Al and O atoms. MnO can promote the transformation of BO and TO to NBO and FO, and the higher the basicity, the more obvious the effect of MnO. Also, the self-diffusion coefficient of each atom increases with the rise of MnO content, which in turn improves the slag viscosity. Yuan et al. [51] revealed that Mn ions tend to interact with BO structural units, thereby modifying the network structure. This behavior is notably different from Mg ions. Mn can serve as charge compensated ions, but its effectiveness is weaker compared to Mg ions. He et al. [99] comparatively analyzed the effect of CaO and MnO on the desulphurization properties of slag using the ab initio MD method. The results showed that MnO is also an ionic oxide, with the same ionic bond between Mn and O atoms, but weaker than Ca-O. A small number of Mn-Mn clusters will be present in the MnO-SiO_2_ melt, while MnO leads to a significant alteration of the desulphurization mechanism. As shown in Figure 8, S will form a stable bonding structure with Mn atoms when incorporated into the liquid MnO-SiO_2_, and the Si-S bond is hardly found in the S-doped MnO-SiO_2_ slag. MnO will result in the decrease of BO and the increase in NBO during desulphurization. MnO will increase the sulphur capacity by increasing the oxygen ion activity and decreasing the activity coefficient of S ions [99].

Na is a typical alkali element, which has been treated as a hazardous element in metallurgical processes. Although its content in molten slag has always been low, its action has been of wide interest. Jiang et al. [64] found that Na_2_O can increase the coordination number of Si-O as well as Al-O in aluminosilicate, with a more pronounced effect on Al-O. Generally, both Si-O and Al-O structures are most stable when they are tetra-coordinated, so the stability of the system decreases successively. Na_2_O has the ability to promote the conversion of BO to NBO, TO, and FO, thereby increasing the diffusion ability and leading to a reduction in viscosity. It indicates that Na_2_O also plays the role of network modifier in the molten slag system, but research has shown that its effect is significantly weaker than that of CaO [44]. In the phosphate glasses system, Na_2_O also plays a role in depolymerizing the network structure of the system and can significantly increase the ratio of NBO oxygen [63]. The number of chemically durable P-O-Fe bridges decreases and they are replaced by P-O-Na. The iron cations will be separated from PO_4_ units and form iron oxides clusters. K_2_O and Na_2_O are typical alkali oxides, and since Na and K belong to the same main group of elements, they are often generally treated as having the same properties. However, they play different roles in terms of microstructural mechanisms [34]. Li et al. [61] comparatively investigated the effects of K_2_O and Na_2_O on the fluidity of coke ash and found that they play opposite roles. K_2_O will lead to an increase in viscosity, while Na_2_O does just the opposite, and yet the effect of both on the microstructure is lacking minimally. Significant differences were however observed in the location of these ions in the oxygen bonding networks. While Na^+^ ions were preferentially located in the bridging/non-bridging oxygen networks, K^+^ ions tended to be present in various oxygen tri-clusters. However, when both basic alkali are present in the slag at the same time, it leads to a sharp BO and NBO increase and a sharp TO decrease [62].

### 4.3. The Effect of Amphoteric Oxide

**Figure 9:**
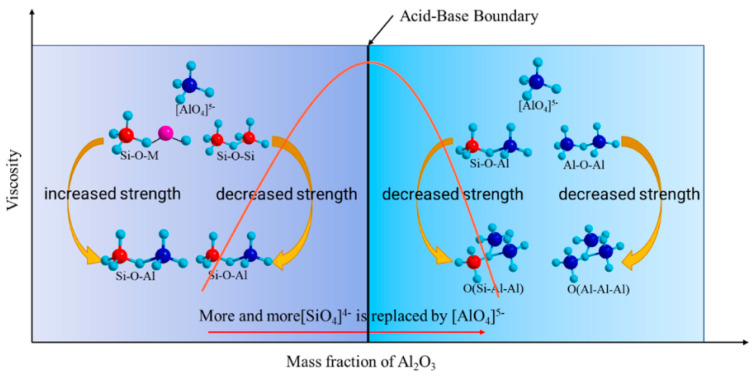
Amphoteric transformation of Al_2_O_3_ in the melt (Reproduced with permission from [36]. Elsevier, 2023).

Amphoteric oxides in different environments can show mutual transformation of both acidic and basic properties. Al_2_O_3_ is a common component of metallurgical molten slag, which in general plays the same role as SiO_2_ and is a constituent unit of the microscopic network structure. However, given the specificity of amphoteric oxides, the active role of Al_2_O_3_ is equally a focus of research. Chen et al. [11] investigated the effect of Al_2_O_3_ on the structure and properties of a CaO-SiO_2_-Al_2_O_3_ system using molecular dynamics simulations. Al_2_O_3_ is considered to be acidic and a network former in the environment with sufficient basic cations. In contrast, Al_2_O_3_ becomes basic with an insufficient basic cations environment, thus providing oxygen atoms and balancing charge. The increase in Al_2_O_3_ concentration leads to a relative shortage of Ca atoms and therefore to an increase in the content of TO and five-coordinated Al, resulting in structural instability. Bi et al. [35] similarly found that Al_2_O_3_ exhibits a significant amphoteric transformation based on the variation in content. When Al_2_O_3_ is relatively low, it mainly exhibits as acidic oxide. In this case, the BO content and polymerization degree of the system increase with the increase in Al_2_O_3_ content. When the content exceeds the critical point, the effect of basic oxides is exerted. It will make the BO content of the system decrease and the growth rate of polymerization degree decrease. Xuan et al. [36] analyzed the effects of the amphoteric transformation process of Al_2_O_3_ on the viscosity and microstructure, based on the results of previous research. As Al_2_O_3_ increases, the coordination number of the basic cations increases, changing from a network modifier to a charge compensation ion. The transition is determined by the charge balancing ability of the slag. The addition of Al_2_O_3_ leads to the replacement of [SiO_4_]^4−^ by [AlO_4_]^5−^, as shown in Figure 9, with a decrease in the contents of Si-O-Si and Si-O-M, and an increase in Si-O-Al, Al-O-Al, and TO. Therefore, the network strength increases first and then decreases, which is consistent with the tendency of viscosity.

TiO_2_ can exhibit amphoteric properties, but there is currently a debate over whether it is truly an amphoteric oxide. Ti ions primarily exist in three forms in the molten slag: [TiO_4_], [TiO_5_], and [TiO_6_]. Most Ti-O-Ti bond angles are 100°, while a small proportion of Ti-O-Ti bond angles vary from 126.67° to 131.64° [57]. Yao et al. [68] found that Si–O–Ti linkages are more favorable than either Si–O–Si or Ti–O–Ti linkages in a CaO-SiO_2_-TiO_2_ system, as Si-O-Ti linkages account for the overwhelming majority of BO groups. Fan and Chen et al. [38,69,70] found that Ti^4+^ ions act as an amphoteric component in slag, i.e., it behaves as a network-forming component with a tetrahedral structure (acidic), or a network-disrupting component with an octahedral structure (basic). Basicity is the key parameter affecting the amphoteric transition of Ti^4+^. As the basicity decreases, the [TiO_4_]^4−^ tetrahedral structure evolves to [TiO_6_]^8−^ octahedral structure, implying that the addition of SiO_2_ favors the formation of [TiO_6_]^8−^ octahedra. Ti-O stretching and Ti-O-Al vibrations can form short-range ordered simple network structures that reduce the effect of SiO_2_ on viscosity. TiO_2_ can reduce system viscosity by replacing SiO_2_, but also consumes BO to form short-range ordered [TiO_4_]^4−^ tetrahedral networks.

In summary, it is shown that amphoteric oxides are capable of both atomic structure network formation and destruction, and the corresponding slag properties will be affected by the amphoteric transition.

## 5. Conclusions and Prospective

In recent years, the development of computers and molecular dynamics has greatly expanded the level and depth of metallurgical process research. The combination of metallurgical physicochemical and atomic simulation can significantly increase the safety, efficiency, and scientificity of the metallurgical experimental process. It is conducive to improving the theory of physicochemistry at the atomic scale and promoting the development of basic research on the properties of oxides in molten slag. Molten slag and its derivatives have been widely valued and studied in the fields of metallurgy, materials, coal chemical industry and earth science. At present, research on the mechanisms of the influence of different natural oxides on the structure of metallurgical slag mainly focuses on the following aspects:

(1) Acidic oxides, represented by SiO_2_ and P_2_O_5_, mainly form [SiO_4_]^4−^ tetrahedra and [PO_4_]^5−^ tetrahedra in molten slag. The [SiO_4_]^4−^ tetrahedra and [PO_4_]^5−^ tetrahedra are interconnected with other polyhedral structures, leading to the formation of complex chain-like or network structures. Acidic oxides often increase the viscosity, liquidus temperature, and enthalpy of molten slag by increasing the degree of polymerization of the its atomic structure.
(2) Basic oxides, represented by CaO and MgO, dissociate into free cations and free oxygen ions in the molten slag. The free cations of basic oxides undergo dynamic equilibrium vibrations around polyhedral structures to compensate for the negative charge overflow. Meanwhile, cations and oxygen ions destroy the network structures of the system during migration and interaction, thereby improving the properties of the melt.
(3) Amphoteric oxides, represented by Al_2_O_3_, possess both acidic and basic oxide properties. When the content of acidic oxides is high, they tend to exhibit the property of basic oxides destroying the network structure. When the content of acidic oxides is low, they generally exhibit the property of forming acidic oxide network structures.

CMD simulations have demonstrated significant capabilities in the field of molten slag research. However, the current composition of metallurgical molten slag has gradually become more complex, and the existing potential functions of CMD can no longer meet the needs of all compositional research. Furthermore, due to the limitations of current force fields, it is difficult for CMD to reproduce the chemical bond properties between different types of atoms in the molten slag, including ionic bonds, covalent bonds, etc., which is also a limitation of CMD. Moreover, current CMD methods can only qualitatively reproduce the regularities of changes in macroscopic properties, and the calculation of macroscopic properties still lacks accuracy. There is an urgent need to develop more accurate potential functions to supplement existing research in depth, aiming to enhance the research of molten slag properties in various scenarios. AIMD is a powerful method for addressing simulations with complex compositions in molten slag, but its computational efficiency limits its applicability to large-scale simulations. However, AIMD can accurately depict the interactions between atoms, characterize the types of chemical bonds between atoms, and elucidate the mechanisms of electron interactions. It serves as an effective complement to CMD methods. Therefore, the synergistic use of CMD and AIMD can address most of the issues in slag simulation. MLMD holds great potential in balancing computational efficiency and accuracy. By ensuring that the dataset used for training the potential functions is diverse enough, the detailed exploration of the structures and properties of molten slag with the coexistence of multiple complex components can be expected. In the future, CMD, AIMD, and MLMD methods will each play crucial roles in different emphasized areas.

## Figures and Tables

**Table 1:** Representative research of CMD in molten slag.

Variable	System	Potential	Temperature	Reference
SiO_2_	SiO_2_	BMH and MORSE	1700–1800 K	[28]
	SiO_2_	Tersoff	300 K and 5000 K	[29]
	SiO_2_-CaO-Al_2_O_3_	BMH	2223 K	[30]
Al_2_O_3_	SiO_2_-CaO-Al_2_O_3_-MgO	BMH	1873 K	[31]
	Na_2_O-Al_2_O_3_, K_2_O-Al_2_O_3_, MgO-Al_2_O_3_, and CaO-Al_2_O_3_,	BMH	2473 K	[32]
	SiO_2_-CaO-Al_2_O_3_-MgO-TiO_2_	BMH	1773 K	[33]
	Na_2_O-Al_2_O_3_-SiO_2_	BMH	4000 K, 3000 K, 2500 K, 2000 K, 1500 K, 1000 K, 750 K, 700 K, 650 K, 600 K, 550 K, 500 K, 30 K	[34]
	SiO_2_-CaO-Al_2_O_3_	BMH	1773 K	[35]
	SiO_2_-CaO-Al_2_O_3_-MgO-FeO	BMH	1873 K	[36]
	SiO_2_-CaO-Al_2_O_3_	BMH	1873 K	[11]
	SiO_2_-CaO-Al_2_O_3_-B_2_O_3_	BMH	1773 K	[37]
	CaO-Al_2_O_3_-TiO_2_	BMH	1873 K	[38]
	SiO_2_-CaO-Al_2_O_3_-MgO	COMPASS	1773 K	[39]
CaO	SiO_2_-CaO-Al_2_O_3_	BMH	1773 K	[40]
	SiO_2_-CaO-Al_2_O_3_-MgO	BMH	1773 K	[41]
	SiO_2_-CaO-Al_2_O_3_-MgO	BMH	1800 K	[42]
	TiO_2_-SiO_2_-MgO-CaO	BMH	1973 K	[43]
	SiO_2_-CaO-Al_2_O_3_-Na_2_O	BMH	1873 K	[44]
	SiO_2_-CaO-Al_2_O_3_	BMH	1773 K	[45]
	SiO_2_-CaO-FeO	BMH	1873 K	[46]
Basicity	SiO_2_-CaO-Al_2_O_3_-FeO	BMH and LJ	1873 K	[1]
	SiO_2_-CaO-FeO-P_2_O_5_	BMH	1673 K	[47]
	SiO_2_-CaO-Al_2_O_3_-MgO	BMH	1873 K	[48]
	SiO_2_-CaO-Al_2_O_3_-MgO-TiO_2_	BMH	1773 K	[33]
	SiO_2_-CaO-B_2_O_3_	BMH	1873 K	[49]
MgO	SiO_2_-CaO-Al_2_O_3_-MgO-TiO_2_	BMH	1773 K	[33]
	SiO_2_-MgO-Al_2_O_3_	BMH	1773 K	[40]
	SiO_2_-CaO-Al_2_O_3_-MgO	BMH	1773 K	[41]
	TiO_2_-SiO_2_-MgO-CaO	BMH	1973 K	[43]
	SiO_2_-MnO-MgO-B_2_O_3_	BMH and LJ	1873 K	[50]
MgO/Al_2_O_3_	CaO-MgO-Al_2_O_3_-SiO_2_	BMH	1773 K	[51]
	CaO-MgO-Al_2_O_3_-SiO_2_	BMH	1773 K	[52]
	CaO-MgO-Al_2_O_3_-SiO_2_	COMPASS	1773 K	[53]
FeO	FeO-TiO_2_	BMH and LJ	1973 K	[54]
	SiO_2_-CaO-FeO	BMH	300 K	[55]
	FeO-TiO_2_-SiO_2_	BMH and LJ	1973 K	[56]
	CaO-SiO_2_-Al_2_O_3_-FeO	BMH	1773 K	[57]
	SiO_2_-CaO-Al_2_O_3_	BMH	1773 K	[45]
	CaO-SiO_2_-Al_2_O_3_-FeO	BMH	1873 K	[58]
	CaO-SiO_2_-FeO-P_2_O_5_	BMH	1673 K	[59]
Na_2_O or K_2_O	CaO-Al_2_O_3_-SiO_2_-Na_2_O(K_2_O)	BMH and MORSE	2273 K	[60]
	CaO-Al_2_O_3_-SiO_2_-Na_2_O-K_2_O	BMH and MORSE	2273 K	[61]
	P_2_O_5_-Fe_2_O_3_-FeO-Na_2_O	BMH	300 K	[62]
	SiO_2_-CaO-Al_2_O_3_-MgO-Na_2_O	BMH and MORSE	1773 K	[63]
	CaO-SiO_2_-Na_2_O	BMH	1773 K	[64]
	SiO_2_-Al_2_O_3_-CaO-Na_2_O	BMH	1873 K	[44]
TiO_2_	CaO-SiO_2_-TiO_2_	BMH	1723 K	[65]
	CaO-SiO_2_-Al_2_O_3_TiO_2_	BMH	1773 K	[66]
	CaO-SiO_2_-TiO_2_	BMH	1873 K	[67]
	FeO-TiO_2_-SiO_2_	BMH and LJ	1973 K	[57]
	CaO-SiO_2_-TiO_2_	BMH	1873 K	[38]
	TiO_2_− MgO-Al_2_O_3_	BMH	1973 K	[68]
	FeO-TiO_2_-B_2_O_3_	BMH	1823 K	[69]
MnO	CaO-SiO_2_-Al_2_O_3_-MnO	BMH and LJ	1773 K	[70]
	SiO_2_-MnO-MgO-B_2_O_3_	BMH and LJ	1873 K	[51]
P_2_O_5_	CaO-P_2_O_5_-SiO_2_	BMH	1673 K	[71]
	V_2_O_5_-P_2_O_5_	BMH	298 K	[72]
	CaO-SiO_2_-P_2_O_5_	BMH	3000 K	[73]
	P_2_O_5_-based binary system	BMH	1873 K	[74]
	SiO_2_-Al_2_O_3_-Na_2_O-P_2_O_5_	Modified Teter	300 K	[75]
	CaO-SiO_2_-P_2_O_5_	BMH	1673 K	[76]
V_2_O_5_	FeO-SiO_2_-V_2_O_3_	BMH	1823 K	[77]
B_2_O_3_	Li_2_O-V_2_O_5_-B_2_O_3_	BMH and MORSE	298 K	[78]
	CaO-Al_2_O_3_-B_2_O_3_	BMH	1973 K	[79]
	SiO_2_-CaO-B_2_O_3_	BMH	1773 K	[35]
	SiO_2_-CaO-Al_2_O_3_-B_2_O_3_	BMH	1773 K	[80]
	SiO_2_-CaO-Al_2_O_3_-B_2_O_3_	BMH	1773 K	[37]
	B_2_O_3_-TiO_2_-CaO-MgO-SiO_2_	BMH	1973 K	[81]
	SiO_2_-CaO-B_2_O_3_	BMH	2573 K	[82]
	CaO-MgO-B_2_O_3_-Al_2_O_3_-SiO_2_	BMH and short order correction	300 K	[83]
CaF	CaO-CaF_2_-SiO_2_	BMH	1773 K	[84]
	CaO-SiO_2_(Al_2_O_3_)-CaF_2_	BMH	1873 K	[85]
	CaO-CaF_2_-SiO_2_	BMH	1823 K	[86]
Li_2_O	CaO-SiO_2_-Al_2_O_3_-Li_2_O	BMH	1673 K	[87]
	CaO-SiO_2_-Al_2_O_3_-Li_2_O	BMH	1673 K	[88]
SiO_2_/Al_2_O_3_ ratio	SiO_2_-MnO-CaF_2_-Al_2_O_3_	BMH	1823 K	[89]
	CaO-SiO_2_-Al_2_O_3_	BMH	2000 K	[90]
	CaO-SiO_2_-Al_2_O_3_-MgO-FeO-TiO_2_-Na_2_O	BMH	1800 K	[91]

**Table 2:** Representative research of AIMD in molten slag.

System	Exchange–Correlation Functionals and Pseudopotential	Temperature	Reference
B_2_O_3_	GGA-PBE, PAW Method	800 K, 2300 K, 3600 K	[94]
CaO-SiO_2_-Al_2_O_3_	GGA-PW91, PAW Method	1773 K	[95]
FeO-SiO_2_-S	GGA-PBE, PAW Method	2000 K	[96]
SiO_2_-P2O_5_-Al_2_O_3_-Na_2_O	GGA-PBE, PAW Method	300 K, 1000 K, 1500, 2000 K	[97]
CaO(MnO)-SiO_2_	GGA-PBE, PAW Method	2000 K	[98]
CaO-Al_2_O_3_-B_2_O_3_	GGA-PBE, GHT-DZVP	1873 K	[99]
Liquid and Amorphous SiO_2_	LDA, ultrasoft PP	300K, 3000 K	[100]
SiO_2_-Al_2_O_3_-CaO	GGA-PBE, PAW Method	2000 K	[101]

Projector-augmented wave (PAW) [102]; Perdew–Burke–Ernzerhof (PBE) [103]; generalized gradient approximation (GGA) [103].

## Data Availability

No new data were created or analyzed in this study. Data sharing is not applicable to this article.

## References

[1] Wu T., Wang Q., Yu C.F., He S.P. (2016). Structural and viscosity properties of CaO–SiO_2_–Al_2_O_3_–FeO slags based on molecular dynamic simulation. J. Non Cryst. Solids.

[2] Ozbay E., Erdemir M., Durmus H.l. (2016). Utilization and efficiency of ground granulated blast furnace slag on concrete properties–A review. Constr. Build. Mater..

[3] Taylor R., Richardson I., Brydson R. (2010). Composition and microstructure of 20-year-old ordinary Portland cement ground granulated blast furnace slag blends containing 0 to 100% slag. Cem. Concr. Res..

[4] Karamanova E., Avdeev G., Karamanov A. (2011). Ceramics from blast furnace slag, kaolin and quartz. J. Eur. Ceram. Soc..

[5] Greeley R., Schneid B.D. (1991). Magma generation on Mars: Amounts, rates, and comparisons with Earth, Moon, and Venus. Science.

[6] Yan Z.-M., Lv X.-W., Li Z.-S. (2022). Physicochemical properties and structure of titania-containing metallurgical slags: A review. J. Iron Steel Res. Int..

[7] Waseda Y., Toguri J.M. (1998). The Structure and Properties of Oxide Melts: Application of Basic Science to Metallurgical Processing.

[8] Liu W., Pang Z., Wang J., Zuo H., Xue Q. (2022). Investigation of viscosity and structure of CaO–SiO_2_–MgO–Al_2_O_3_–BaO–B_2_O_3_ slag melt. Ceram. Int..

[9] Qiu G., Miao D., Wei X., Bai C., Li X. (2022). Effect of MgO/Al_2_O_3_ and CaO/SiO_2_ on the Metallurgical Properties of CaO–SiO_2_–Al_2_O_3_–MgO–TiO_2_ Slag. J. Non Cryst. Solids.

[10] Yao L., Ren S., Wang X., Liu Q., Dong L., Yang J., Liu J. (2016). Effect of Al_2_O_3_, MgO, and CaO/SiO_2_ on viscosity of high alumina blast furnace slag. Steel Res. Int..

[11] Chen Y., Pan W., Jia B., Wang Q., Zhang X., Wang Q., He S. (2021). Effects of the amphoteric behavior of Al_2_O_3_ on the structure and properties of CaO–SiO_2_–Al_2_O_3_ melts by molecular dynamics. J. Non Cryst. Solids.

[12] Pang Z., Lv X., Jiang Y., Ling J., Yan Z. (2020). Blast furnace ironmaking process with super-high TiO_2_ in the slag: Viscosity and melting properties of the slag. Metall. Mater. Trans. B.

[13] Yan H.-j., Liu L., Zhuang J.-c., Zhou P., Zhou C.Q. (2018). Molecular Dynamics Simulation of Carbon Effect on the Thermal Physical Properties of the Molten Iron. ISIJ Int..

[14] Yan W., Chen W., Yang Y., Lippold C., McLean A. (2016). Evaluation of B_2_O_3_ as replacement for CaF_2_ in CaO–Al_2_O_3_ based mould flux. Ironmak. Steelmak..

[15] Kim G.H., Sohn I. (2019). Effect of CaF_2_, B_2_O_3_ and the CaO/SiO_2_ mass ratio on the viscosity and structure of B_2_O_3_ containing calcium silicate based melts. J. Am. Ceram. Soc..

[16] Liao J., Li J., Wang X., Zhang Z. (2012). Influence of TiO_2_ and basicity on viscosity of Ti bearing slag. Ironmak. Steelmak..

[17] Park H., Park J.Y., Kim G.H., Sohn I. (2012). Effect of TiO_2_ on the viscosity and slag structure in blast furnace type slags. Steel Res. Int..

[18] Shankar A., Görnerup M., Lahiri A., Seetharaman S. (2007). Estimation of viscosity for blast furnace type slags. Ironmak. Steelmak..

[19] Lv X., Lv X., Qiu J., Liu M. (2017). Viscosity and Structure Evolution of Slag in Ferronickel Smelting Process from Laterite. J. Min. Metall. Sect. B Metall..

[20] Min D.J., Tsukihashi F. (2017). Recent advances in understanding physical properties of metallurgical slags. Met. Mater. Int..

[21] Zhou C., Li J., Wang S., Zhao J., Ai L., Chen Q., Chen Q., Zhao D. (2023). Development of Molecular Dynamics and Research Progress in the Study of Slag. Materials.

[22] Finnis M., Sinclair J. (1984). A simple empirical N-body potential for transition metals. Philos. Mag. A.

[23] Vanommeslaeghe K., Hatcher E., Acharya C., Kundu S., Zhong S., Shim J., Darian E., Guvench O., Lopes P., Vorobyov I. (2010). CHARMM general force field: A force field for drug-like molecules compatible with the CHARMM all-atom additive biological force fields. J. Comput. Chem..

[24] Cornell W.D., Cieplak P., Bayly C.I., Gould I.R., Merz K.M., Ferguson D.M., Spellmeyer D.C., Fox T., Caldwell J.W., Kollman P.A. (1995). A second generation force field for the simulation of proteins, nucleic acids, and organic molecules. J. Am. Chem. Soc..

[25] Chenoweth K., Van Duin A.C., Goddard W.A. (2008). ReaxFF reactive force field for molecular dynamics simulations of hydrocarbon oxidation. J. Phys. Chem. A.

[26] Tersoff J. (1988). New empirical approach for the structure and energy of covalent systems. Phys. Rev. B.

[27] Sun H. (1998). COMPASS: An ab initio force-field optimized for condensed-phase applications overview with details on alkane and benzene compounds. J. Phys. Chem. B.

[28] Hoang V.V. (2007). Molecular dynamics simulation of amorphous SiO_2_ nanoparticles. J. Phys. Chem. B.

[29] Nath S.D. (2013). Study of the effect of sizes on the structural properties of SiO_2_ glass by molecular dynamics simulations. J. Non Cryst. Solids.

[30] Li K., Bouhadja M., Khanna R., Zhang J., Liu Z., Zhang Y., Yang T., Sahajwalla V., Yang Y., Barati M. (2016). Influence of SiO_2_ reduction on the local structural order and fluidity of molten coke ash in the high temperature zone of a blast furnace. A molecular dynamics simulation investigation. Fuel.

[31] Liu Y., Bai C., Lv X., Wei R. (2015). Molecular dynamics simulation on the influence of Al_2_O_3_ on the slag structure at 1873 K. Mater. Today Proc..

[32] Wu T., Wang Q., Yao T., He S. (2016). Molecular dynamics simulations of the structural properties of Al_2_O_3_-based binary systems. J. Non Cryst. Solids.

[33] Yuelin Q., Hao L., Yanhua Y. (2018). Structure evolution of blast furnace slag with high Al_2_O_3_ Content and 5 mass% TiO_2_ via molecular dynamics simulation and fourier transform infrared spectroscopy. Metall. Res. Technol..

[34] Zhao Y., Du J., Qiao X., Cao X., Zhang C., Xu G., Liu Y., Peng S., Han G. (2020). Ionic self-diffusion of Na_2_O–Al_2_O_3_–SiO_2_ glasses from molecular dynamics simulations. J. Non Cryst. Solids.

[35] Bi Z., Li K., Jiang C., Zhang J., Ma S. (2021). Effects of amphoteric oxide (Al_2_O_3_ and B_2_O_3_) on the structure and properties of SiO_2_–CaO melts by molecular dynamics simulation. J. Non Cryst. Solids.

[36] Xuan W., Zhang Y. (2023). Exploration of the amphoteric transition of Al_2_O_3_ on melt structure and viscosity of silicate slag. Ceram. Int..

[37] Bi Z., Li K., Jiang C., Zhang J., Ma S., Sun M., Wang Z., Li H. (2021). Performance and transition mechanism from acidity to basicity of amphoteric oxides (Al_2_O_3_ and B_2_O_3_) in SiO_2_–CaO–Al_2_O_3_–B_2_O_3_ system: A molecular dynamics study. Ceram. Int..

[38] Chen Y., Yang J., Zhang X., Wang Q., Wang Q., He S. (2022). Amphoteric behavior of component and microstructure feature on CaO-Al_2_O_3_-TiO_2_ ternary melt by molecular dynamics simulation. Comput. Mater. Sci..

[39] Liu J., Yu Y., Kong W., Wang Q., He Z., Yang X. (2022). Effect of Al_2_O_3_ on the molten slag microstructure information by molecular dynamics simulation and experimental analysis. J. Non Cryst. Solids.

[40] Jiang C., Li K., Zhang J., Qin Q., Liu Z., Liang W., Sun M., Wang Z. (2018). The effect of CaO (MgO) on the structure and properties of aluminosilicate system by molecular dynamics simulation. J. Mol. Liq..

[41] Jiang C., Li K., Zhang J., Liu Z., Niu L., Liang W., Sun M., Ma H., Wang Z. (2019). The effect of CaO and MgO on the structure and properties of coal ash in the blast furnace: A molecular dynamics simulation and thermodynamic calculation. Chem. Eng. Sci..

[42] Zhao H., Li J., Yang S., Liu J., Liu W. (2021). Molecular dynamics study of structural properties of refining slag with various CaO/Al_2_O_3_ ratios. Minerals.

[43] Fan H., Wang R., Duan H., Chen D., Xu Z. (2021). Structural and transport properties of TiO_2_–SiO_2_–MgO–CaO system through molecular dynamics simulations. J. Mol. Liq..

[44] Gao L., Liu X., Bai J., Kong L., Bai Z., Li W. (2021). Structure and flow properties of coal ash slag using ring statistics and molecular dynamics simulation: Role of CaO/Na_2_O in SiO_2_–Al_2_O_3_–CaO–Na_2_O. Chem. Eng. Sci..

[45] Ma S., Li K., Zhang J., Jiang C., Bi Z., Sun M., Wang Z., Li H. (2021). The effects of CaO and FeO on the structure and properties of aluminosilicate system: A molecular dynamics study. J. Mol. Liq..

[46] Gu C., Lyu Z., Bao Y. (2022). Size-dependent dissolution behavior of CaO in the CaO–SiO_2_–FeO slag system: A molecular dynamics study. J. Mol. Liq..

[47] Diao J., Ke Z., Jiang L., Zhang Z., Zhang T., Xie B. (2017). Structural properties of molten CaO–SiO_2_–P_2_O_5_–FeO system. High. Temp. Mater. Process..

[48] Liu Y., Lv X., Li B., Bai C. (2018). Relationship between structure and viscosity of CaO–SiO_2_–MgO–30.00 wt% Al_2_O_3_ slag by molecular dynamics simulation with FT-IR and Raman spectroscopy. Ironmak. Steelmak..

[49] Sadaf S., Wu T., Zhong L., Liao Z.Y., Wang H.C. (2020). Effect of basicity on the structure, viscosity and crystallization of CaO–SiO_2_-B_2_O_3_ based mold fluxes. Metals.

[50] Yuan H., Wang Z., Zhang Y., Wang C. (2023). Roles of MnO and MgO on structural and thermophysical properties of SiO_2_–MnO–MgO-B_2_O_3_ welding Fluxes: A molecular dynamics study. J. Mol. Liq..

[51] Jiang C., Li K., Zhang J., Qin Q., Liu Z., Sun M., Wang Z., Liang W. (2018). Effect of MgO/Al_2_O_3_ ratio on the structure and properties of blast furnace slags: A molecular dynamics simulation. J. Non Cryst. Solids.

[52] Jiang C., Li K., Zhang J., Qin Q., Liu Z., Liang W., Sun M., Wang Z. (2019). Molecular dynamics simulation on the effect of MgO/Al_2_O_3_ ratio on structure and properties of blast furnace slag under different basicity conditions. Metall. Mater. Trans. B.

[53] Kong W.-G., Liu J.-H., Yu Y.-W., Hou X.-M., He Z.-J. (2021). Effect of w (MgO)/w (Al_2_O_3_) ratio and basicity on microstructure and metallurgical properties of blast furnace slag. J. Iron Steel Res. Int..

[54] Fan H., Chen D., Liu P., Duan H., Huang Y., Long M., Liu T. (2018). Structural and transport properties of FeO–TiO_2_ system through molecular dynamics simulations. J. Non Cryst. Solids.

[55] Siakati C., Macchieraldo R., Kirchner B., Tielens F., Peys A., Seveno D., Pontikes Y. (2020). Unraveling the nano-structure of a glassy CaO-FeO-SiO_2_ slag by molecular dynamics simulations. J. Non Cryst. Solids.

[56] Fan H., Wang R., Xu Z., Duan H., Chen D. (2021). Structural and transport properties of FeO–TiO_2_–SiO_2_ systems: Insights from molecular dynamics simulations. J. Non Cryst. Solids.

[57] Ma S., Li K., Zhang J., Jiang C., Sun M., Li H., Wang Z., Bi Z. (2021). Structural Characteristics of CaO–SiO_2_–Al_2_O_3_–FeO Slag with Various FeO Contents Based on Molecular Dynamics Simulations. JOM.

[58] Ma S., Ren S., Li K., Zhang J., Jiang C., Bi Z., Sun M. (2022). The Effects of FeO and Fe_2_O_3_ on the Structure and Properties of Aluminosilicate System: A Molecular Dynamics Study. JOM.

[59] Yang H., Liu Y., Wan X., Zhang T.-a., Lin S., Wang K. (2023). The Effect of FeO on Transport Properties of Dephosphorization Slag from Microstructure: A Molecular Dynamics Simulation Study. Trans. Indian. Inst. Met..

[60] Li K., Khanna R., Bouhadja M., Zhang J., Liu Z., Su B., Yang T., Sahajwalla V., Singh C.V., Barati M. (2017). A molecular dynamic simulation on the factors influencing the fluidity of molten coke ash during alkalization with K_2_O and Na_2_O. Chem. Eng. J..

[61] Li K., Khanna R., Zhang J., Bouhadja M., Sun M., Barati M., Liu Z., Singh C.V. (2017). Molecular dynamics investigation on coke ash behavior in the high-temperature zones of a blast furnace: Influence of alkalis. Energy Fuels.

[62] Goj P., Stoch P. (2018). Molecular dynamics simulations of P_2_O_5_-Fe_2_O_3_-FeO-Na_2_O glasses. J. Non Cryst. Solids.

[63] Jiang C., Zhang H., Xiong Z., Chen S., Li K., Zhang J., Liang W., Sun M., Wang Z., Wang L. (2020). Molecular dynamics investigations on the effect of Na_2_O on the structure and properties of blast furnace slag under different basicity conditions. J. Mol. Liq..

[64] Zhang X., Liu C., Jiang M. (2021). Effect of Na Ions on Melt Structure and Viscosity of CaO–SiO_2_–Na_2_O by Molecular Dynamics Simulations. ISIJ Int..

[65] Zhang S., Zhang X., Bai C., Wen L., Lv X. (2013). Effect of TiO2 content on the structure of CaO–SiO_2_–TiO_2_ system by molecular dynamics simulation. ISIJ Int..

[66] Zhang S., Zhang X., Peng H., Wen L., Qiu G., Hu M., Bai C. (2014). Structure analysis of CaO–SiO_2_–Al_2_O_3_–TiO_2_ slag by molecular dynamics simulation and FT-IR spectroscopy. ISIJ Int..

[67] Yao T.-H., He S.-P., Wu T., Wang Q. (2017). Molecular dynamics simulations of microstructural properties of CaO–SiO_2_–TiO_2_ fluorine-free slag systems. Ironmak. Steelmak..

[68] Fan H., Zhu Y., Xu Z., Wang R. (2022). Structure of the TiO_2_−MgO–Al_2_O_3_ system: Insights from molecular dynamics simulations. J. Non Cryst. Solids.

[69] Kim Y., Nam C., Kim S., Jeon H. (2021). Rheological properties and structure of molten FeO-TiO_2_-B2O_3_ ilmenite smelting slag. J. Non Cryst. Solids.

[70] Ma S., Li K., Zhang J., Jiang C., Bi Z., Sun M., Wang Z. (2021). Effect of MnO content on slag structure and properties under different basicity conditions: A molecular dynamics study. J. Mol. Liq..

[71] Fan G., Diao J., Jiang L., Zhang Z., Xie B. (2015). Molecular dynamics analysis of the microstructure of the CaO–P_2_O_5_–SiO_2_ slag system with varying P_2_O_5_/SiO_2_ ratios. Mater. Trans..

[72] Saiko I., Saetova N., Raskovalov A., Il’ina E., Molchanova N., Kadyrova N. (2020). Hopping conductivity in V_2_O_5_–P_2_O_5_ glasses: Experiment and non-constant force field molecular dynamics. Solid. State Ion..

[73] Nguyen M.A., Nguyen V.H. (2023). Structural Properties of Liquid CaO–SiO_2_–P_2_O_5_ System. VNU J. Sci. Math. Phys..

[74] Du Y., Yuan Y., Li L., Long M., Duan H., Chen D. (2021). Insights into structure and properties of P_2_O_5_-based binary systems through molecular dynamics simulations. J. Mol. Liq..

[75] Zhao Y., Du J., Cao X., Zhang C., Xu G., Qiao X., Liu Y., Peng S., Han G. (2021). A modified random network model for P_2_O_5_–Na_2_O–Al_2_O_3_–SiO_2_ glass studied by molecular dynamics simulations. RSC Adv..

[76] Sun H., Yang J., Zhang R., Xu L. (2024). Insight into the structure and transport properties of CaO-SiO_2_-P_2_O_5_ system during the phosphorus enrichment process: A molecular dynamics simulation. J. Non Cryst. Solids.

[77] Zhang Z., Xie B., Zhou W., Diao J., Li H.-Y. (2016). Structural characterization of FeO–SiO_2_–V_2_O_3_ slags using molecular dynamics simulations and FT-IR spectroscopy. ISIJ Int..

[78] Saetova N., Raskovalov A., Antonov B., Denisova T., Zhuravlev N. (2020). Structural features of Li_2_O–V_2_O_5_–B_2_O_3_ glasses: Experiment and molecular dynamics simulation. J. Non Cryst. Solids.

[79] Feng X., Yao W., Li J. (2021). Effect of B_2_O_3_ on the structure of CaO–Al_2_O_3_–B_2_O_3_ ternary melts: A molecular dynamics simulation. J. Non Cryst. Solids.

[80] Bi Z., Li K., Jiang C., Zhang J., Ma S., Sun M., Wang Z., Li H. (2021). Effects of B_2_O_3_ on the structure and properties of blast furnace slag by molecular dynamics simulation. J. Non Cryst. Solids.

[81] Fan H., Wang R., Xu Z., Duan H., Chen D. (2021). The effect of B2O3 on the structure and properties of titanium slag melt by molecular dynamics simulations. J. Mater. Res. Technol..

[82] Zhang X., Liu C., Jiang M. (2022). Effect of B_2_O_3_ on the Melt Structure and Viscosity of CaO–SiO_2_ System. Steel Res. Int..

[83] Yang R., Zhang Y., Zu Q., Huang S., Zhang L., Deng L., Zeng H. (2023). Molecular dynamics simulations study on structure and properties of CaO–MgO–B_2_O_3_–Al_2_O_3_–SiO_2_ glasses with different B_2_O_3_/MgO. J. Non Cryst. Solids.

[84] Asada T., Yamada Y., Ito K. (2008). The estimation of structural properties for molten CaO–CaF_2_–SiO_2_ system by molecular dynamics simulations. ISIJ Int..

[85] Zhang X., Liu C., Jiang M. (2020). Effect of Fluorine on Melt Structure for CaO–SiO_2_–CaF_2_ and CaO–Al_2_O_3_–CaF_2_ by Molecular Dynamics Simulations. ISIJ Int..

[86] Zhang X., Liu C., Jiang M., Sun J., Zheng X. (2022). The Relationship Between Composition and Structure: The Influence of Different Methods of Adding CaF_2_ on the Melt Structure of CaO–SiO_2_ Slags by Molecular Dynamics Simulations. J. Sustain. Metall..

[87] Jia B., Li M., Yan X., Wang Q., He S. (2019). Structure investigation of CaO–SiO_2_–Al_2_O_3_–Li_2_O by molecular dynamics simulation and Raman spectroscopy. J. Non Cryst. Solids.

[88] Jiao S., Guo P., Chen F., Min Y., Liu C. (2024). Molecular Dynamics Analysis of the Structural Behavior of Aluminum Ion in the Slag of CaO–SiO_2_–Al_2_O_3_–Li_2_O System. Steel Res. Int..

[89] Wang Z., Li Z., Zhong M., Li Z., Wang C. (2023). Elucidating the effect of Al_2_O_3_/SiO_2_ mass ratio upon SiO_2_-MnO-CaF_2_-Al_2_O_3_-based welding fluxes: Structural analysis and thermodynamic evaluation. J. Non Cryst. Solids.

[90] Zheng K., Zhang Z., Yang F., Sridhar S. (2012). Molecular dynamics study of the structural properties of calcium aluminosilicate slags with varying Al_2_O_3_/SiO_2_ ratios. ISIJ Int..

[91] Xu B., Cao Y., Wang Z., Du P., Long Y. (2022). Molecular Dynamics Study on the Effect of SiO_2_/Al_2_O_3_ Mass Ratio on the Structural Properties and Viscosity of Molten Fused Red Mud. Minerals.

[92] Marx D., Hutter J. (2000). Ab initio molecular dynamics: Theory and implementation. Mod. Methods Algorithms Quantum Chem..

[93] Iftimie R., Minary P., Tuckerman M.E. (2005). Ab initio molecular dynamics: Concepts, recent developments, and future trends. Proc. Natl. Acad. Sci. USA.

[94] Scherer C., Schmid F., Letz M., Horbach J. (2019). Structure and dynamics of B2O3 melts and glasses: From ab initio to classical molecular dynamics simulations. Comput. Mater. Sci..

[95] Sajid M., Bai C., Yu W., You Z., Tan M., Aamir M. (2020). First principle study of electronic structural and physical properties of CaOSiO_2_Al_2_O_3_ ternary slag system by using Ab Initio molecular and lattice dynamics. J. Non Cryst. Solids.

[96] He X., Ma S., Wang L., Dong H., Chou K. (2021). An Ab Initio Molecular Dynamics Simulation of Liquid FeO–SiO_2_ Silicate System with Sulfur Dissolving. Metall. Mater. Trans. B.

[97] Qian Y., Song B., Jin J., Prayogo G.I., Utimula K., Nakano K., Maezono R., Hongo K., Zhao G. (2022). Ab initio molecular dynamics simulation of structural and elastic properties of SiO_2_–P2O_5_–Al_2_O_3_–Na_2_O glass. J. Am. Ceram. Soc..

[98] He X., Ma S., Wang L., Dong H., Chou K. (2022). Comparison of desulfurization mechanism in liquid CaO–SiO_2_ and MnO–SiO_2_: An ab initio molecular dynamics simulation. J. Alloys Compd..

[99] Zhang C., Kong Y.-Q., Wu T., Lei J., Wang H.-C. (2022). First-principles study on microstructure of CaO–Al_2_O_3_–B_2_O_3_ slag. J. Mol. Liq..

[100] Sarnthein J., Pasquarello A., Car R. (1995). Structural and electronic properties of liquid and amorphous si o 2: An ab initio molecular dynamics study. Phys. Rev. Lett..

[101] Jiang C., Li K., Barati M., Guo P., Danaei A., Liang W., Zhang J. (2023). The interaction mechanism between molten SiO_2_–Al_2_O_3_–CaO slag and graphite with different crystal orientations: Experiment and Ab initio molecular dynamics simulation. Ceram. Int..

[102] Blöchl P.E. (1994). Projector augmented-wave method. Phys. Rev. B.

[103] Perdew J.P., Burke K., Ernzerhof M. (1996). Generalized gradient approximation made simple. Phys. Rev. Lett..

[104] Gasteiger J., Zupan J. (1993). Neural networks in chemistry. Angew. Chem. Int. Ed. Engl..

[105] Senior A.W., Evans R., Jumper J., Kirkpatrick J., Sifre L., Green T., Qin C., Žídek A., Nelson A.W., Bridgland A. (2020). Improved protein structure prediction using potentials from deep learning. Nature.

[106] Dral P.O. (2020). Quantum chemistry in the age of machine learning. J. Phys. Chem. Lett..

[107] Zhang L., Han J., Wang H., Car R., Weinan E. (2018). Deep potential molecular dynamics: A scalable model with the accuracy of quantum mechanics. Phys. Rev. Lett..

[108] Sun Y., Tan M., Li T., Li J., Shang B. (2022). Study on the structural properties of refining slags by molecular dynamics with deep learning potential. J. Mol. Liq..

[109] Jiang T., Wang S., Guo Y.F., Chen F., Zheng F.Q. (2016). Effects of Basicity and MgO in Slag on the Behaviors of Smelting Vanadium Titanomagnetite in the Direct Reduction-Electric Furnace Process. Metals.

[110] Wei Zhang K.Z. (2011). jiating Rao, Weiguo Fu and Mansheng Chu, Discussion on standardization of Melt-property Temperature of slag. J. IRon Steel Res..

[111] Liang H., Chu M., Feng C., Tang J., Liu Z., Wang W. (2018). Optimisation study and affecting mechanism of CaO/SiO_2_ and MgO on viscous behaviours of titanium-bearing blast furnace slag. Ironmak. Steelmak..

[112] Gao Y., Bian L., Liang Z. (2015). Influence of B_2_O_3_ and TiO_2_ on viscosity of titanium-bearing blast furnace slag. Steel Res. Int..

[113] Gao Y.-M., Wang S.-B., Hong C., Ma X.-J., Yang F. (2014). Effects of basicity and MgO content on the viscosity of the SiO_2_–CaO–MgO–9 wt% Al_2_O_3_ slag system. Int. J. Miner. Metall. Mater..

[114] Kim H., Kim W.H., Sohn I., Min D.J. (2010). The effect of MgO on the viscosity of the CaO–SiO_2_–20 wt% Al_2_O_3_–MgO slag system. Steel Res. Int..

[115] Jiao K.X., Zhang J.L., Wang Z.Y., Chen C.L., Liu Y.X. (2017). Effect of TiO_2_ and FeO on the viscosity and structure of blast furnace primary slags. Steel Res. Int..

[116] Xu R.Z., Zhang J.L., Han W.X., Chang Z.Y., Jiao K.X. (2018). Effect of BaO and Na_2_O on the viscosity and structure of blast furnace slag. Ironmak. Steelmak..

[117] Ye G., Yang J., Zhang R., Yang W., Sun H. (2021). Behavior of phosphorus enrichment in dephosphorization slag at low temperature and low basicity. Int. J. Miner. Metall. Mater..

[118] Schwitalla D.H., Guhl S., Körner J., Laabs M., Bai J., Meyer B. (2021). Meta-study on the effect of P_2_O_5_ on single phase slag viscosity and the effect of P_2_O_5_ induced liquid phase immiscibility on dispersion viscosity. Fuel.

[119] Xia Y., Li J., Fan D., Hou G. (2019). Effects of interfacial oxygen potential and slag phase changing during slag formation process on dephosphorization behavior. ISIJ Int..

[120] Sun Y., Zheng K., Liao J., Wang X., Zhang Z. (2014). Effect of P2O5 addition on the viscosity and structure of titanium bearing blast furnace slags. ISIJ Int..

[121] Zhang S., Li H., Ran M., Jiang Z., Zheng L., Feng H., Yu J., Dai Y. (2022). Effect of Al_2_O_3_ on Viscosity and Refining Ability of High Basicity Slag for Heat-resistant Austenitic Stainless Steel. ISIJ Int..

[122] Xu R.Z., Zhang J.L., Wang Z.Y., Jiao K.X. (2017). Influence of Cr_2_O_3_ and B_2_O_3_ on viscosity and structure of high alumina slag. Steel Res. Int..

[123] Cherry J., Davies H., Mehmood S., Lavery N., Brown S., Sienz J. (2015). Investigation into the effect of process parameters on microstructural and physical properties of 316L stainless steel parts by selective laser melting. Int. J. Adv. Manuf. Technol..

[124] Zhang J., Lv X., Yan Z., Qin Y., Bai C. (2016). Desulphurisation ability of blast furnace slag containing high Al_2_O_3_ and 5 mass% TiO_2_ at 1773 K. Ironmak. Steelmak..

[125] Shen F., Jiang X., Wu G., Wei G., Li X., Shen Y. (2006). Proper MgO addition in blast furnace operation. ISIJ Int..

[126] Liu W., Zuo H. (2021). Effect of MnO and substituting CaO with BaO on the desulfurization ability of blast furnace slag. Metall. Mater. Trans. B.

[127] Ju J., Liu W., Xing X., Wang J., An J. (2019). Sulphide capacity of CaO–SiO_2_–8% MgO–Al_2_O_3_–BaO slags ranging from 0% to 5% in BaO. Metall. Res. Technol..

[128] Park J.H., Park G.-H. (2012). Sulfide capacity of CaO–SiO_2_–MnO–Al_2_O_3_–MgO Slags at 1873 K. ISIJ Int..

[129] Talapaneni T., Yedla N., Sarkar S. (2018). Study on desulfurization capacity of high alumina blast furnace slag at 1773 K using slag-metal equilibrium technique. Metall. Res. Technol..

[130] Liu W., Xing X., Zuo H. (2020). Effect of TiO_2_ on viscosity and sulfide capacity of blast furnace slag containing barium. ISIJ Int..

[131] Xu C.-y., Wang C., Xu R.-Z., Zhang J.-l., Jiao K.-X. (2021). Effect of Al_2_O_3_ on the viscosity of CaO–SiO_2_–Al_2_O_3_–MgO–Cr_2_O_3_ slags. Int. J. Miner. Metall. Mater..

[132] Wang H., Li G., Zhu X., Zhao Y. (2012). Research on Activities of CaO–SiO_2_–B_2_O_3_(Al_2_O_3_, Fe_2_O_3_) Slag System. Appl. Mech. Mater..

[133] Lai F., Yao W., Li J. (2020). Effect of B_2_O_3_ on structure of CaO–Al_2_O_3_–SiO_2_–TiO_2_–B_2_O_3_ glassy systems. ISIJ Int..

